# The emergence of highly pathogenic avian influenza H5N1 in dairy cattle: implications for public health, animal health, and pandemic preparedness

**DOI:** 10.1007/s10096-025-05147-z

**Published:** 2025-05-14

**Authors:** Mohamed Kamel, Sami Aleya, Wesam Taher Almagharbeh, Lotfi Aleya, Mohamed M. Abdel-Daim

**Affiliations:** 1 Department of Medicine and Infectious Diseases, Faculty of Veterinary Medicine, Cairo University, Giza, 11221 Egypt; 2 Faculty of Medicine, Université de Bourgogne Franche-Comté, Hauts-du-Chazal, Besançon, 25030 France; 3 Medical and Surgical Nursing Department, Faculty of Nursing, University of Tabuk, Tabuk, 7149 Saudi Arabia; 4 Laboratoire de Chrono-Environnement, Université de Bourgogne Franche-Comté, UMR CNRS 6249, La Bouloie, Besançon, 25030 France; 5 Department of Pharmaceutical Sciences, Pharmacy Program, Batterjee Medical College, Jeddah, 21442 Saudi Arabia; 6 Pharmacology Department, Faculty of Veterinary Medicine, Suez Canal University, Ismailia, 41522 Egypt

**Keywords:** HPAI H5N1, Dairy cattle, Zoonosis, Viral adaptation, Milk safety, One health, Surveillance, Biosecurity, Public health risk

## Abstract

**Background:**

The 2024 outbreak of Highly Pathogenic Avian Influenza (HPAI) H5N1 in U.S. dairy cattle represents a significant change in the behavior of zoonotic influenza viruses. Previously, H5N1 was primarily an avian pathogen with limited infection in mammals. The emergence of clade 2.3.4.4b H5N1 in dairy herds across multiple states reveals the virus’s adaptation to mammalian hosts and highlights its potential for zoonotic transmission, raising important concerns for public health, veterinary medicine, and agriculture.

**Results:**

The virus demonstrated unique tropism for mammary tissue, with high viral loads detected in milk. Genomic analysis identified mutations that enhance binding to mammalian receptors and facilitate systemic spread. Viral RNA was found in raw milk, posing food safety risks; however, standard pasteurization effectively inactivated the virus. Epidemiological data indicate the outbreak likely began with spillover from wild birds or contaminated fomites, followed by efficient local transmission within herds. Forty-one human cases linked to infected dairy operations were confirmed. The outbreak caused significant economic losses due to decreased milk production and trade restrictions. Although human-to-human transmission remains low, the zoonotic risk requires urgent attention.

**Conclusion:**

The 2024 HPAI H5N1 outbreak in U.S. dairy cattle highlights critical gaps in surveillance, biosecurity, and coordination across sectors. A One Health approach integrating veterinary, public health, and environmental efforts is essential. Recommendations include improved surveillance, stringent biosecurity measures, occupational safety protocols, focused research on viral evolution, and investment in diagnostics and vaccines. These actions are vital to reduce risks, protect public health, and ensure the sustainability of the dairy industry against future zoonotic influenza threats.

## Introduction

### Background on highly pathogenic avian influenza (HPAI) H5N1

Highly Pathogenic Avian Influenza (HPAI) H5N1, first identified in 1996 in Guangdong, China, has evolved into a significant global health concern [1]. The virus, classified under the A/goose/Guangdong/1/96 lineage, is characterized by its high mortality rates in infected birds and its ability to infect a wide range of avian and mammalian species, including humans, cats, dogs, sea lions, pigs, cattle, goats, and many others [2–5]. Since its emergence, HPAI H5N1 has undergone significant genetic diversification through mutation, reassortment, and natural selection, leading to the emergence of distinct lineages and clades [6, 7].

The global impact of HPAI H5N1 is significant, affecting animal health, economic stability, and public health [8–11]. The virus has caused significant mortality in wild bird populations and led to the culling of millions of poultry worldwide, resulting in substantial economic losses for the agricultural sector. As of May 22, 2024, the World Health Organization (WHO) has reported 891 confirmed human cases of H5N1 infection across 24 countries, with 463 deaths, resulting in a high case fatality rate of 52% since 2003 [12].

HPAI H5N1 primarily spreads through direct contact with infected birds or contaminated environments. The transmission dynamics are influenced by migratory patterns of wild birds, which serve as reservoirs for the virus. Since its emergence, the virus has spread to numerous countries across Asia, Europe, and Africa, causing devastating outbreaks in poultry populations [13–15].

The economic impact of HPAI H5N1 extends beyond the poultry industry, significantly affecting populations through increased egg and poultry product prices [16]. Outbreaks often lead to mass culling of birds, disrupting supply chains and reducing the availability of eggs and poultry meat in the market [17]. This scarcity drives up costs, placing a disproportionate burden on low-income households, for whom eggs and poultry are essential and affordable protein sources [18]. In developing countries, where food insecurity is already prevalent, these price surges exacerbate nutritional deficiencies and economic hardships. Furthermore, higher egg prices strain household budgets globally, forcing consumers to reallocate spending or reduce protein consumption, thereby impacting overall dietary quality [19]. These cascading effects highlight the critical need for effective outbreak management to stabilize markets and protect vulnerable populations [16, 20].

The emergence of clade 2.3.4.4b H5N1 has further complicated the epidemiological landscape. This strain has demonstrated increased virulence and adaptability, leading to spillover events into mammals, including domestic livestock [21–24]. The virus’s ability to evolve, adapt to new hosts, and cross species barriers underscores the need for ongoing surveillance, research, and preparedness efforts. In an unprecedented development, HPAI H5N1 was detected in US dairy cattle for the first time in March 2024 [24, 25]. As of November 2024, the virus has been confirmed in over 508 dairy herds across 16 US states, with California, Colorado, Idaho, Michigan, and Texas being the most affected [26].

Affected dairy cows often exhibit several clinical signs. Firstly, there is a sudden decrease in feed intake and rumination [27, 28]. This is usually accompanied by a marked drop in milk production. The appearance of the milk may become abnormal, taking on a yellow, thickened, colostrum-like quality [27]. Additionally, the consistency of the manure can vary significantly [27, 29]. Lastly pneumonia and mastitis (necrotizing mastitis), can occur [30]. Importantly, intra-mammary infection stimulates an immune defense that fully protects the mammary gland from reinfections, inhibiting clinical signs, replication, and shedding [31].

Diagnostic tests have confirmed the presence of viral RNA in milk and nasal swabs, with characteristic lesions observed in mammary tissue [32]. In additions, after aerosol inoculation of four 1-year-old Holstein heifers with 2 mL of 1 × 10⁶ HPAI H5N1 virus, viral RNA was repeatedly detected in oropharyngeal, ocular, and saliva samples from one calf, while the other three showed only sporadic, non-consecutive positives [33]. Viral RNA was occasionally detected at low levels in blood, nasal secretions, and urine, but was consistently absent in faeces. At post-mortem (4 DPI), high levels of viral RNA were found in the inoculated hindquarter mammary tissue and detected in the inguinal/supra-mammary lymph node, spleen, liver, and mesenteric lymph node, but not detected in the mediastinal lymph node, lung, or kidney. Additionally, viral RNA was identified in air samples collected during routine husbandry activities [34]. Genetic sequencing has identified the virus as H5N1 clade 2.3.4.4b, the same lineage causing widespread outbreaks in wild birds and poultry [27, 32].

Epidemiological investigations suggest the initial introduction of HPAI H5N1 into dairy herds likely occurred via contact with infected wild birds, followed by limited local spread and onward horizontal transmission between cattle [27, 35, 36]. The high levels of viral RNA detected in milk indicate this may be an important route of shedding [37].

The detection of HPAI H5N1 in dairy cattle raises several concerns: (i) viral adaptation to mammals, increasing the risk of enhanced mammalian transmissibility; (ii) zoonotic potential, with 65 human cases linked to exposure to infected dairy cattle or poultry operations as of April 2025 [38]; (iii) food safety concerns, although extensive testing has found no evidence of infectious virus in pasteurized milk or other dairy products; and (iv) significant economic impacts on the dairy industry.

The spillover of HPAI H5N1 into US dairy cattle represents a significant development in the evolution and ecology of zoonotic influenza viruses, with important implications for public health, veterinary medicine, and pandemic preparedness. This outbreak challenges long-standing views on host susceptibility and underscores the need for a One Health approach in managing the risks posed by zoonotic influenza viruses.

## The unprecedented nature of the spillover event

### Historical context: HPAI H5N1 as a primarily avian pathogen

HPAI H5N1 viruses have long been recognized as primarily affecting wild birds and poultry. The earliest known HPAI H5N1 virus was isolated from a chicken farm outbreak in Scotland in 1959 [39–41]. However, it was not until 1996 that the precursor virus of the currently circulating Asian HPAI H5N1 lineage was detected in southern China, causing an outbreak in domestic geese with an unusually high 40% morbidity rate [1].

The A/goose/Guangdong/1/96 (Gs/Gd) lineage emerged through ongoing mutation, reassortment, and natural selection of low pathogenic avian influenza (LPAI) H5N1 viruses circulating in wild birds [7, 42, 43]. This lineage subsequently diverged into distinct clades and expanded into multiple avian reservoir hosts, with domestic and aquatic poultry serving as the primary reservoirs in the late 1990s and early 2000s [44, 45].

From 1996 to 2003, HPAI H5N1 outbreaks were largely confined to poultry populations in East and Southeast Asia [46]. The role of wild migratory birds in the global spread of HPAI H5N1 became apparent in 2005 when the virus was detected in wild waterfowl at Qinghai Lake in China [47]. This marked a key turning point in the spread of the virus beyond Asia.

Throughout much of its evolutionary history, HPAI H5N1 has behaved as a primarily avian pathogen, with only sporadic zoonotic transmission to humans and other mammalian species. From 2003 to July 2023, 868 human cases and 457 deaths from HPAI H5N1 were documented by the WHO [48]. While the virus can cause severe disease in humans, sustained human-to-human transmission has not been observed during this period [49].

### Previous instances of HPAI H5N1 in mammals and their limited scope

Prior to the current outbreak, HPAI H5N1 infections had been documented in a variety of mammalian species, including wild carnivores, domestic cats and dogs, and humans [50, 51]. However, these infections were generally isolated incidents, often associated with direct contact with infected birds or their carcasses. The viruses did not demonstrate the ability to spread readily between mammals, and the infections were typically self-limiting.

Several factors contributed to the limited scope of HPAI H5N1 infections in mammals: (I) earlier strains were not well-adapted to mammalian hosts, reducing efficient transmission; (II) rapid identification and containment of poultry outbreaks limited mammalian exposure; (III) genetic barriers, such as the absence of specific mutations necessary for efficient replication in mammals, restricted the spread; and (IV) ecological factors also played a role, as limited interaction between infected birds and susceptible mammals reduced transmission opportunities.

### The scale and severity of the current outbreak in US dairy cattle

The current outbreak of HPAI H5N1 in US dairy cattle represents a significant departure from the historical pattern of HPAI H5N1 infections in mammals. Since its initial detection in March 2024, the virus has spread rapidly across multiple states, infecting at least 875 dairy herds in 16 states [52]. The virus has been confirmed in dairy herds across California, Colorado, and Idaho, and additional cases have been reported in Michigan, Texas, Iowa, and other states.

Key features of the current outbreak include: (I) widespread transmission, as the virus efficiently replicates and spreads among cattle, both within and between dairy farms [53]; (II) unique tropism, with the virus showing a strong affinity for the mammary gland of infected cows, leading to high viral loads in the milk [54]; (III) economic impact, as the mortality rate among infected cows remains low at around 2%, but substantial economic losses occur due to a dramatic reduction in milk production, with yields dropping by 10–20% for 7–10 days [2]; (IV) public health concerns, highlighted by the detection of a human case in a Texas dairy farm worker [55], athough the risk of H5N1 transmission from cattle to humans is currently considered low; and (V) food safety issues, with the presence of H5N1 virus fragments in raw milk from affected farms raising questions about milk safety, although pasteurization effectively kills the virus, ensuring the safety of the commercial milk supply [54, 55].

In response to the outbreak, the USDA has implemented a series of measures to control the spread of the virus and support affected producers. These include movement restrictions, comprehensive testing protocols, and financial assistance worth approximately $200 million [56]. The unprecedented nature of this outbreak, marked by its scale, severity, and the virus’s adaptation to a new mammalian host, underscores the need for continued vigilance, research, and coordinated response efforts to mitigate its impact and prevent future zoonotic disease emergencies.

## Implications of HPAI H5N1 in dairy cattle: transmission, adaptation, and zoonotic potential

The outbreak of HPAI H5N1 in US dairy cattle has not only raised significant concerns about transmission routes and potential adaptation to mammalian hosts but has also highlighted broader implications for other livestock species, such as swine. The swine industry, in particular, is regarded as a critical concern due to pigs’ susceptibility to both avian and human influenza strains, making them potential “mixing vessels” for the emergence of novel reassortant viruses [57]. The incursion of H5N1 into swine populations could amplify the risk of zoonotic spillover and interspecies transmission, given the close contact between pigs and humans in many farming systems. Additionally, the environmental contamination noted in dairy cattle outbreaks raises concerns about cross-species transmission pathways, which may include shared water sources or feed supplies between cattle and swine operations [57, 58]. The following section explores the specific transmission pathways from avian reservoirs to cattle in greater detail.

### Transmission routes from avian reservoirs to cattle

The unprecedented 2024 outbreak of HPAI H5N1 in US dairy cattle has raised significant concerns about the potential routes of transmission from avian reservoirs to cattle populations [27, 55]. While the exact mechanisms are still under investigation, several possible pathways have been proposed based on epidemiological evidence and preliminary research findings:

#### Wild bird introductions

One of the primary hypotheses is that migratory wild birds, which are natural reservoirs for avian influenza viruses, initially introduced H5N1 into cattle herds [37]. The HPAI H5N1 virus causing the cattle outbreak is closely related to strains circulating in North American wild birds [8, 59]. Waterfowl and shorebirds can shed high levels of virus in their feces, potentially contaminating pastures, feed, or water sources that cattle come into contact with [8]. However, there is currently no direct genomic or epidemiologic evidence confirming wild birds as the source of cattle infections. The possibility cannot be ruled out though, especially given the high environmental viral load from concurrent poultry outbreaks.

#### Fomite transmission

Another potential route is mechanical transmission via fomites - objects or materials that can carry infection, such as equipment, vehicles, clothing, or bedding. The HPAI H5N1 virus could be introduced to cattle farms by contaminated equipment shared between poultry and cattle operations or carried on the clothing/footwear of personnel moving between facilities. Specific risk factors that have been proposed include shared farm staff, uncleaned vehicles and equipment moving between premises, and frequent on- and off-farm visitations [60]. Virus-contaminated poultry litter, which can be used as a cattle feed supplement or fertilizer in some areas, is another possible fomite. However, fomite transmission from poultry farms to cattle operations is still considered an unlikely route.

#### Bovine-to-Bovine transmission

Once HPAI H5N1 is introduced into a cattle herd, epidemiological evidence suggests the virus can spread from cow to cow [61, 62]. Animal movements, particularly transport of infected cattle between farms, appears to be a major risk factor for between-herd transmission. Within a herd, the virus may transmit between cows via contaminated milking equipment, on the hands/clothing of milking personnel, or through infectious milk droplets [2, 27]. Cows can shed virus in milk from infected udders, even in the absence of respiratory symptoms [2]. Suckling calves can also be probably infected by consuming raw milk from H5N1-positive dams as demonstrated previously in cats and mice [36, 63]. The strains of dairy cattle did exhibit an ability to shed via the air, raising questions about the transmission route of the H5N1 virus [64]. This observation is particularly noteworthy since the shedding patterns align with the transmission dynamics seen in ferret-to-ferret studies [64], suggesting that understanding airborne transmission in cattle could play a crucial role in determining the virus’s potential to adapt and spread.

While cow-to-cow transmission has been observed following transport of infected animals [36, 37], the exact mechanisms of transmission remain unclear. Sampling of infected cattle found virus mainly in the milk and mammary glands rather than respiratory secretions [37, 65, 66]. More studies are needed to fully characterize transmission mechanisms and efficiency.

#### Other potential routes

Contaminated feed and water sources are additional possibilities for H5N1 introduction to cattle, but direct evidence is currently lacking [61]. The presence of H5 RNA in wastewater from California in March 2024, occurring before outbreaks in dairy herds in other states, raises concerns about the potential unnoticed spread of H5N1 among regional dairy cattle or poultry [67]. Respiratory exposure from nearby infected poultry flocks has also been suggested but would likely require a high viral dose and close proximity to overcome the host species barrier [2, 27].

**Fig. 1:**
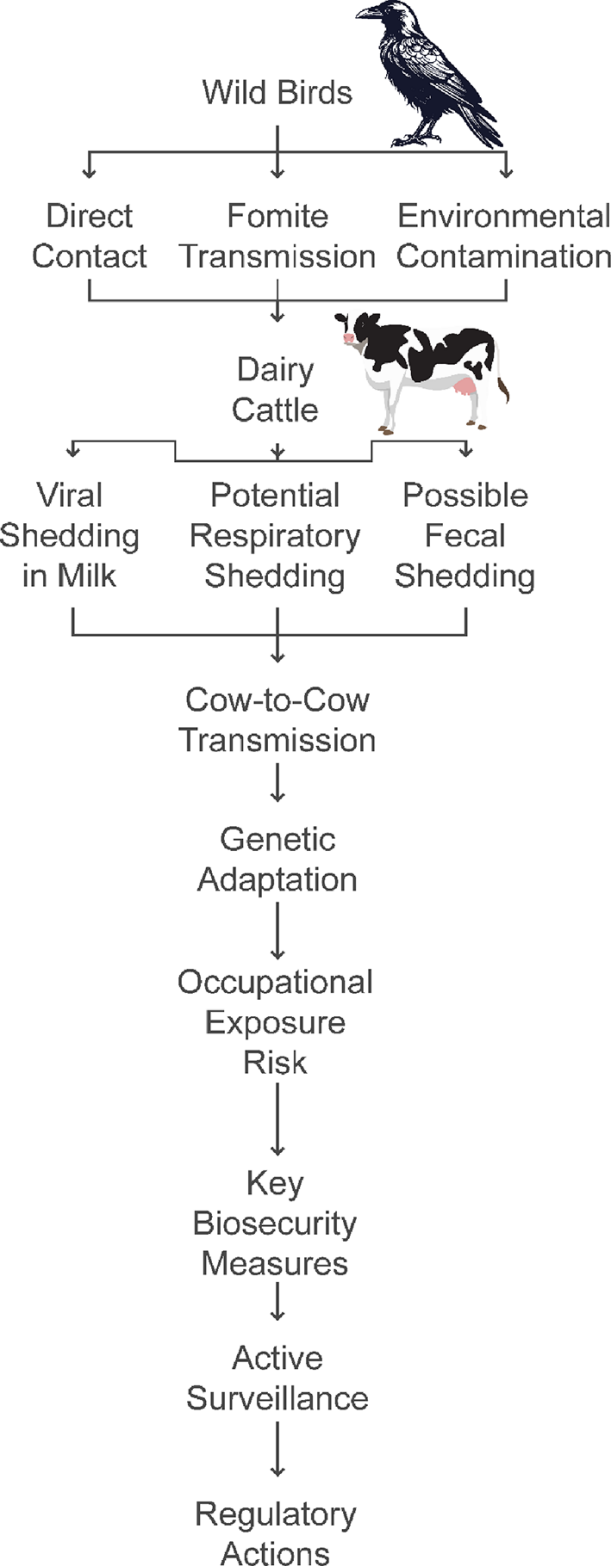
Transmission Pathways of Viral Infections from Wild Birds to Dairy Cattle. This figure shows viral transmission pathways from wild birds to dairy cattle, highlighting shedding routes and biosecurity measures

Therefore, the most plausible routes based on current data appear to be: (1) initial “spillover“ from wild birds or fomites from poultry farms, followed by (2) between-farm spread via animal movements, and (3) within-farm transmission through contaminated milking processes (Fig. 1). However, significant knowledge gaps remain. Ongoing research and surveillance are critical to better understand the transmission dynamics of HPAI H5N1 from avian reservoirs to cattle populations. Identifying the key routes will be essential for implementing effective prevention and control strategies.

### Molecular basis for host adaptation and increased transmissibility in mammals

The molecular basis for host adaptation and increased transmissibility of HPAI H5N1 in mammals is a multifaceted process that involves specific genetic mutations and receptor interactions [48, 68]. Understanding these molecular mechanisms is crucial for developing effective control strategies and preparing for potential zoonotic spillover events that could pose significant public health risks [69].

#### Genomic characterization of bovine H5N1 viruses

Genetic analysis of HPAI H5N1 viruses isolated from infected dairy cattle has revealed that they belong to a new genotype, B3.13, within the 2.3.4.4b clade [70]. These viruses share nearly identical genome sequences and have undergone two reassortment events since 2023 [24]. Notably, the bovine H5N1 viruses exhibit critical mutations in the hemagglutinin (HA), matrix protein 1 (M1), and non-structural protein 1 (NS1) genes [24].

The HA gene of bovine H5N1 viruses contains amino acid residues 137 A, 158 N, and 160 A, which have been associated with increased binding affinity to human-type receptors [70, 71]. The M1 gene harbors residues 30D, 43 M, and 215 A, while the NS1 gene possesses residues 42 S, 103 F, and 106 M, all of which have been linked to enhanced virulence [24]. However, the bovine H5N1 viruses lack critical mutations in the polymerase basic 2 (PB2) and PB1 genes that are known to enhance virulence or adaptation to mammalian hosts [24].

More recently, a distinct genotype, D1.1, has been reported in dairy cattle, indicating ongoing viral evolution in this host [55, 72]. The detection of multiple HPAI H5N1 genotypes in cattle underscores the dynamic nature of the virus and highlights the importance of continued genomic surveillance to assess emerging public health risks [70, 73].

#### Receptor binding specificity and tissue tropism

Experimental studies have demonstrated that bovine H5N1 viruses exhibit a unique dual receptor binding specificity, with the ability to bind to both avian-type (α2,3-linked sialic acid) and human-type (α2,6-linked sialic acid) receptors [74]. This dual receptor binding specificity differs from previously circulating HPAI H5N1 viruses and suggests that bovine H5N1 viruses may have the capacity to infect cells in the human upper respiratory tract [74]. The A/bovine/OH/B24OSU-432/2024 strain preferentially binds to α2,3-sialoside “avian-type” receptors and retains specific hemagglutinin amino acids linked to this receptor specificity [74].

In animal models, bovine H5N1 viruses have shown a propensity to spread systemically beyond the respiratory tract, including to the mammary glands, eyes, and muscles [68]. This systemic spread has been observed following both respiratory infection and oral ingestion of the virus. The ability of bovine H5N1 viruses to replicate in the mammary glands raises concerns about the potential for transmission through raw milk consumption.

### Transmission potential and public health implications

While bovine H5N1 viruses possess molecular features that may facilitate infection and transmission in mammals, experimental studies in ferrets have indicated limited respiratory droplet transmission [68]. In a study where infected ferrets were housed next to uninfected ferrets, only one out of four exposed ferrets seroconverted without detectable virus, suggesting inefficient airborne transmission [68].

However, the detection of H5N1 in the milk of infected cows and the ability of the virus to cause severe disease in mice and ferrets following oral ingestion highlight the potential risk of transmission through the consumption of raw or unpasteurized milk [68, 75]. The spillover of bovine H5N1 viruses to other mammalian species, including cats and humans, underscores the need for continued surveillance and monitoring of the virus’s evolution [27, 76].

The identification of a human case associated with cattle exposure, where the PB2 E627K mutation was detected, emphasizes the potential for rapid evolution of the virus post-infection and the importance of vigilant monitoring to assess public health threats [77–79]. While the current risk to the general public remains low, proactive surveillance, early detection, and collaboration among health agencies and stakeholders are crucial to effectively manage and contain the outbreak.

### Comparison to other zoonotic influenza viruses (e.g., H5N8, H7N9)

The HPAI H5N1 virus has garnered significant attention due to its potential for zoonotic transmission and severe pathogenicity in humans. Since its emergence in the late 1990s, H5N1 has been associated with a high case fatality rate, with approximately 52% of reported human infections resulting in death [48]. In contrast, other zoonotic influenza viruses, such as H5N8 and H7N9, present different epidemiological profiles and pathogenicity [80, 81].

H5N8, while also a highly pathogenic strain, has primarily affected avian populations and has shown limited human infections, with no sustained human-to-human transmission reported [82, 83]. This distinction is crucial as it highlights the varying degrees of risk associated with different subtypes of avian influenza, emphasizing the need for tailored surveillance and response strategies.

H7N9, on the other hand, has demonstrated a more concerning pattern of zoonotic transmission. First identified in humans in 2013, H7N9 has resulted in over 1,500 reported cases, with a case fatality rate of approximately 40% [84, 85]. Unlike H5N1, H7N9 has shown a greater capacity for human adaptation, with several mutations identified that enhance its ability to bind to human-type sialic acid receptors [86, 87]. This receptor binding affinity is a critical factor in determining the transmissibility of influenza viruses between species. The ability of H7N9 to infect humans more readily than H5N1 (four times) raises significant public health concerns, particularly in regions with high poultry density and close human-animal interactions [88].

The differences in host adaptation and transmissibility among these viruses underscore the importance of understanding the molecular mechanisms that govern their pathogenicity. For instance, mutations in the hemagglutinin (HA) and neuraminidase (NA) proteins of H7N9 have been linked to increased virulence and the ability to evade host immune responses [89, 90]. In contrast, while H5N1 has retained its avian receptor preference, recent studies indicate that it may still possess the potential for adaptation in mammals, as evidenced by its recent spillover into dairy cattle [68]. This adaptability, combined with the ongoing evolution of these viruses, necessitates continuous monitoring and research to assess the risks they pose to public health and to develop effective prevention strategies [91–93].

## Implications of HPAI H5N1 in dairy cattle: milk safety, public health, and industry response

### Understanding the threat of HPAI H5N1 in milk: Raw vs. Pasteurized

#### Assessing the risk of HPAI H5N1 transmission through raw and pasteurized milk

The emergence of HPAI H5N1 in dairy cattle has raised significant concerns regarding the potential transmission of the virus through milk products [36]. Studies have shown that raw milk from infected cows can contain high viral loads, with H5N1 being shed at levels ranging from 10^4^ to 10^8.8^TCID50/mL in milk samples, and up to 10^7.8^ TCID50/mL in mammary gland tissues [27]. This indicates efficient viral replication in milk-secreting epithelial cells. The virus has been found to remain viable in refrigerated raw milk for at least 5 weeks with infectious virus detected for up to 8 weeks in both clinical samples and experimentally spiked milk, demonstrating its potential for prolonged infectivity [94].

Experimental studies have further highlighted the risks associated with raw milk consumption. When fed raw milk from an infected cow, mice developed illness and had detectable virus in respiratory and other organs [63]. These findings underscore the potential infectivity of raw milk containing HPAI H5N1, posing a risk not only to farm workers but also to consumers of unpasteurized dairy products.

The detection of H5N1 genetic material in approximately 20% of retail pasteurized milk samples has complicated the risk assessment [95]. While this suggests that the virus may not be viable post-pasteurization, its presence indicates potential contamination during the milking process.

#### Effectiveness of pasteurization

Pasteurization is a critical control measure designed to inactivate pathogens in milk, and its effectiveness against HPAI H5N1 has been well-demonstrated [95]. Standard high-temperature short-time (HTST) pasteurization, which involves heating milk to at least 72 °C for 15 s, significantly reduces viral titers, often to undetectable levels, even in milk samples spiked with high virus concentrations [95]. Specifically, heating to 63 °C for at least 5 min completely inactivated the virus [95]. Additionally, heating to 72 °C for at least 30 s is supposed to eliminate infectious viruses, though some residual virus was detected at 15–20 s.

**Fig. 2:**
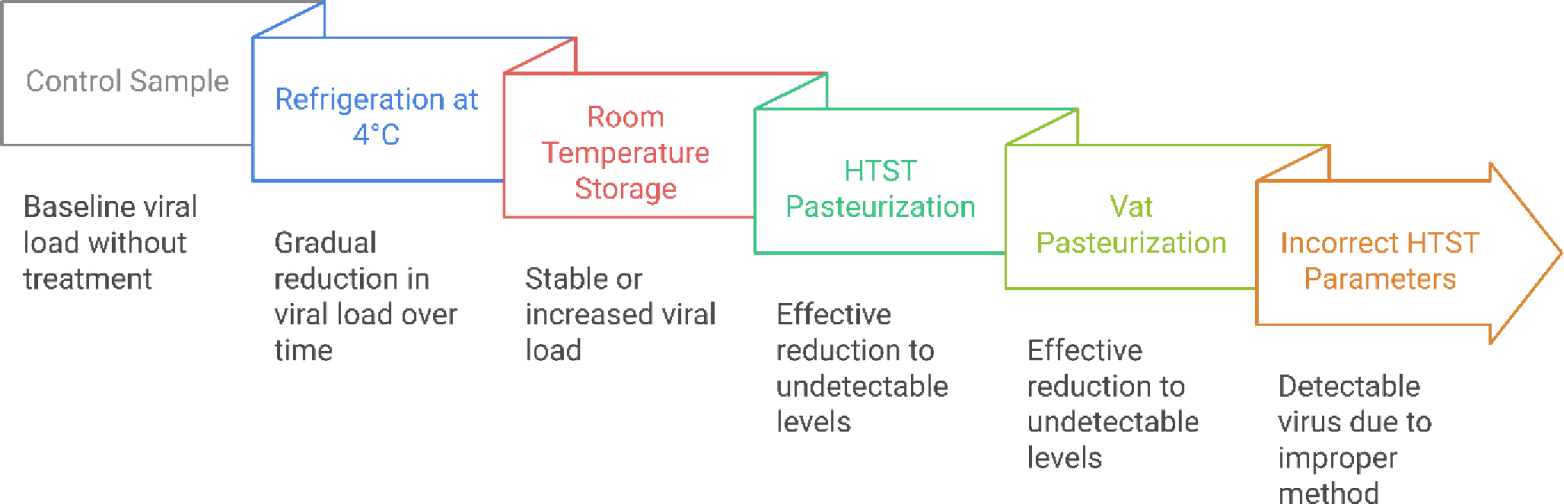
Impact of Treatments on HPAIV Viral Load in Raw Milk

However, some experimental studies have reported residual infectious virus after pasteurization under specific conditions, highlighting the importance of adhering to established pasteurization protocols [63, 96]. The variability in pasteurization efficacy may be influenced by factors such as initial viral load, the presence of fat globules, and the specific pasteurization equipment used, which may not replicate laboratory conditions (Fig. 2).

This figure illustrates the effects of various treatments on the viral load of HPAIV in raw milk. The control sample represents the baseline viral load without treatment. Subsequent treatments are shown, including refrigeration at 4 °C, which leads to a gradual reduction in viral load over time, and room temperature storage, where viral load remains stable or increases. High-Temperature Short-Time (HTST) pasteurization and vat pasteurization are effective in reducing viral loads to undetectable levels. However, using incorrect HTST parameters can result in detectable virus due to improper method application.

#### Ongoing concerns and recommendations

Despite the effectiveness of pasteurization, the risk of HPAI H5N1 transmission through raw milk remains a concern, particularly in light of the recent outbreaks in dairy cattle. The persistence of the virus in raw milk, coupled with the potential for cross-contamination during milking and processing, necessitates stringent biosecurity measures on farms [97]. Regulatory agencies have recommended that raw milk not be consumed, and ongoing surveillance of both raw and pasteurized milk products is essential to ensure public safety.

**Fig. 3:**
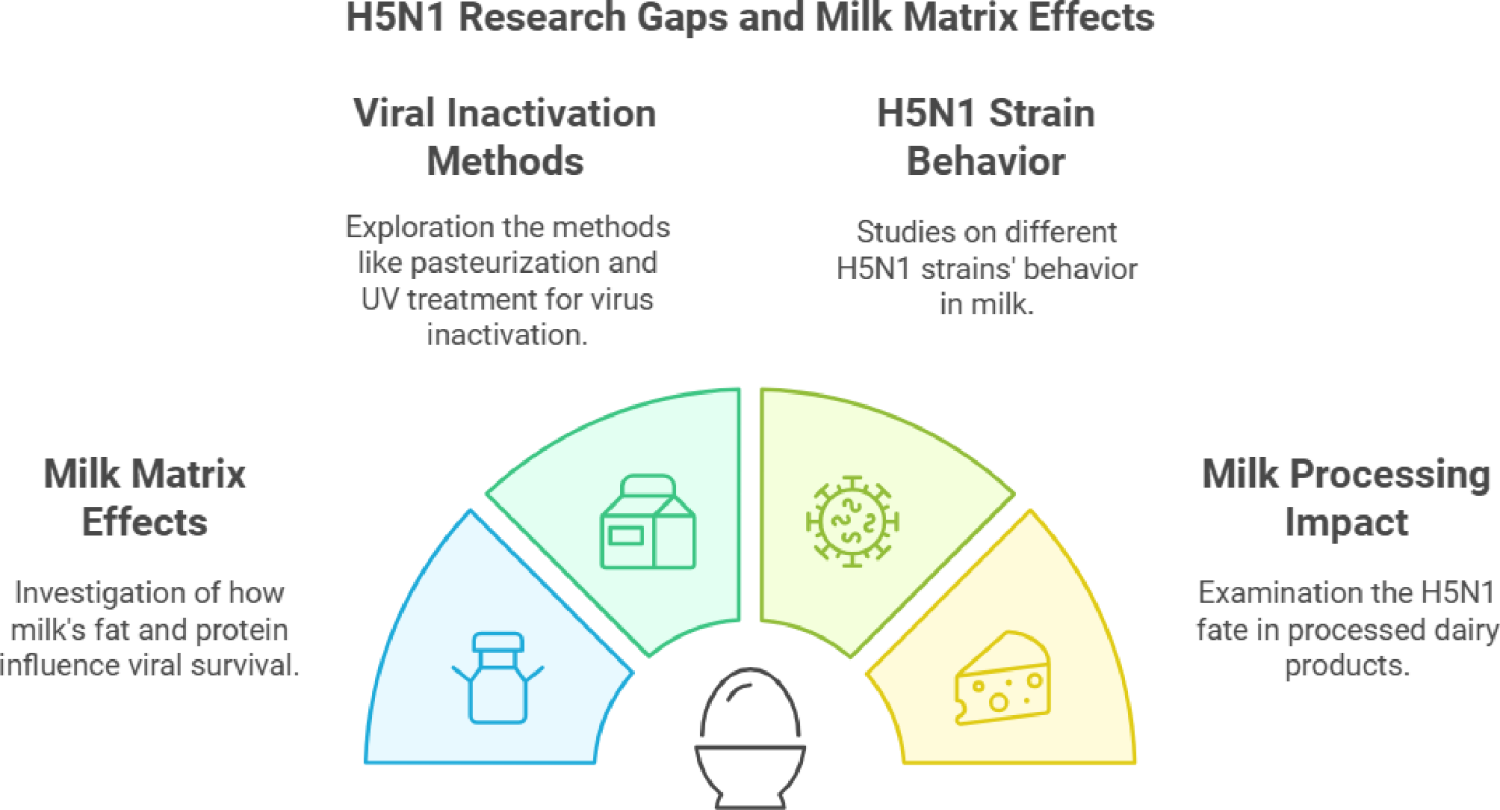
Research Gaps in H5N1 Virus Inactivation and Milk Matrix Effects

Some uncertainties remain regarding the effectiveness of pasteurization for certain products like colostrum or powdered milk, unlike regular liquid milk, produced through freeze-drying. As the situation evolves, further research is needed to fully understand the dynamics of HPAI H5N1 transmission through dairy products and to develop effective strategies to mitigate the associated risks (Fig. 3).

This figure summarizes key research gaps in H5N1 virus inactivation and milk matrix effects.

### Potential for occupational exposure and human-to-human transmission

The outbreak of HPAI H5N1 in dairy cattle has significantly increased the risk of occupational exposure among farm workers [98] and raised concerns about the potential for human-to-human transmission.

#### Occupational exposure risks

Dairy farm workers are at heightened risk due to their close and frequent interactions with infected animals, particularly during milking, feeding, and handling of raw milk. The milking environment, characterized by the use of shared equipment and the potential for splatter, creates multiple opportunities for exposure to the virus [5, 54, 97, 99, 100]. The lack of stringent personal protective equipment (PPE) protocols in many dairy operations exacerbates this risk, as workers often do not wear masks or eye protection, increasing the likelihood of infection through mucosal surfaces [83, 101]. Poultry farms exhibit higher PPE usage than dairy operations, primarily favoring gloves over eye or face protection, yet PPE implementation is often inadequate and inconsistent [55, 102]. Environmental contamination poses an additional risk for occupational exposure for both dairy and poultry workers [103, 104]. The virus can persist in the farm environment, creating indirect exposure risks through contaminated surfaces, equipment, feed, or water sources [97, 99].

#### Human cases and symptoms

As of April 2024, there have been at least 24 confirmed human cases of HPAI H5N1 infection associated with exposure to sick or infected dairy cattle in the United States, with cases reported in Texas, Michigan, and Colorado [105]. The symptoms in human cases have primarily been mild, with conjunctivitis being the most common presentation [54, 83]. Notably, the first confirmed human case in Texas involved a dairy farm worker who exhibited conjunctivitis after direct exposure to infected cows [54]. The virus has a human case fatality rate of 59–66%, indicating its potential for severe health emergencies [106, 107]. H5N1’s neurovirulence, with its ability to invade neural tissues, may lead to unpredictable cognitive and neurological effects if a human pandemic occurs [108].

#### Potential for human-to-human transmission

While the primary mode of transmission for HPAI H5N1 has historically been through direct contact with infected birds or their environments, the recent spillover into mammals, including dairy cattle, suggests a potential shift in transmission dynamics [68, 109]. This development has important implications for occupational risk: both dairy and poultry workers are now considered high-risk groups for HPAI H5N1 exposure [103]. Dairy workers may be exposed through direct contact with infected cattle, handling raw milk or milking equipment, and working in contaminated environments, as the virus can be present in milk, feces, and other secretions [68, 110]. Handling raw milk or working with milking devices before pasteurization is particularly concerning, as infectious virus has been detected at high levels in these materials [37, 110]. Additionally, contaminated surfaces and equipment, as well as environmental contamination from feces, further increase the risk for dairy workers. Poultry workers remain at high risk due to direct handling of infected or dead birds and contaminated materials, particularly during slaughter and processing tasks [103, 111]. The risk is greatest when workers are exposed without appropriate personal protective equipment (PPE) or experience breaches in PPE [103]. Both dairy and poultry workers may inadvertently contribute to cross-species and inter-facility transmission, especially when personnel, vehicles, or equipment are shared between premises or when biosecurity protocols are lacking [103]. Notably, the movement of infected animals and shared labor between dairy and poultry operations can facilitate virus spread, posing risks not only to workers but also to their households and surrounding communities [103]. These overlapping risks underscore the need for rigorous biosecurity practices, appropriate use of PPE, and ongoing surveillance to protect workers and limit the potential for zoonotic transmission as the virus continues to adapt within mammalian hosts [103, 110].

Although sustained human-to-human transmission has not been documented, the presence of the virus in human cases linked to occupational exposure raises alarms about the possibility of mutations that could facilitate such transmission. The genetic adaptations observed in H5N1 viruses, particularly those that enhance receptor binding and replication in mammalian tissues, could increase the risk of efficient human transmission if the virus were to acquire the necessary mutations.

#### Transmission studies and risk assessment

Transmission studies have offered valuable insights into the potential for human-to-human spread of HPAI H5N1 from the dairy cattle outbreak. Experiments with ferrets showed that bovine H5N1 viruses did not transmit efficiently via respiratory droplets, indicating that the current risk of human-to-human spread remains low [68]. However, one study reported limited (33%) respiratory droplet transmission in ferrets of an HPAI H5N1 virus isolated from an infected farm worker in Texas [68].

As of July 2024, the Centers for Disease Control and Prevention (CDC) still considers the H5N1 bird flu human health risk for the US general public to be low [103]. However, the agency emphasizes that people with close or prolonged, unprotected exposures to infected animals or contaminated environments are at greater risk of infection [103].

#### Recommendations and ongoing efforts

To mitigate these risks, the CDC has updated interim recommendations for worker protection and the use of PPE in dairy farm settings [112, 113]. Enhanced biosecurity practices, restricted access, and proper disinfection protocols are recommended for affected farms [55, 112].

Ongoing surveillance efforts are critical to monitor the spread of HPAI H5N1 and detect any potential human cases. The CDC is conducting enhanced and targeted surveillance in affected areas and has implemented monitoring and testing strategies for exposed persons. The agency recommends that individuals working in high-risk environments be vigilant for symptoms and undergo regular health screenings to detect any potential infections early.

### The need for enhanced surveillance and biosecurity measures in the dairy industry

The rapid spread and impact of HPAI H5N1 in dairy cattle highlights the urgent need for enhanced surveillance and strengthened biosecurity measures in the dairy industry. Enhanced surveillance involves several key strategies. (I) Routine sampling and testing, including bulk tank milk testing, are crucial for quickly detecting HPAI incursions in dairy herds. Programs like the Dairy Herd Status Program enable producers to monitor their herds through weekly bulk milk testing. (II) Movement testing, which involves testing individual cattle before interstate movement, helps track the virus’s spread. (III) Product testing ensures food safety by monitoring virus presence in the supply chain. (IV) The USDA has initiated testing of culled dairy cow carcasses and milk products for HPAI as part of comprehensive surveillance and safety monitoring efforts. (V) Mandatory reporting of HPAI cases and positive test results is vital for swift response and containment. Producers and veterinarians must remain vigilant and promptly report any suspected infections.

Strengthening biosecurity measures is equally important. (I) Reducing wild bird exposure by limiting their access to cattle and their environment, including feed and water sources, is essential. Implementing bird-proofing measures and deterrents can further reduce risks. (II) Strict biosecurity protocols for personnel and equipment moving between farms are necessary. This includes cleaning and disinfection of vehicles, using dedicated clothing and footwear, and restricting unnecessary visitors to prevent farm-to-farm transmission. (III) Animal management practices, such as isolating sick or exposed animals and enforcing 21-day quarantines for new or returning cattle, help limit potential spread within herds. (IV) Regular cleaning and disinfection of facilities, equipment, and water troughs eliminate the virus from the environment, with particular attention to removing bird feces. (V) The proper use of PPE by farm workers, especially during high-risk activities like milking, is crucial. Providing training on correct PPE usage and disposal is essential. (VI) Robust herd health management programs, including regular veterinary check-ups and prompt treatment of sick animals, are necessary. Vaccination strategies should be considered if approved vaccines become available. (VII) Maintaining detailed records of animal movements, health status, and farm visitors aids in contact tracing if an outbreak occurs. (VIII) Ongoing education and training for farm workers on HPAI symptoms, biosecurity measures, and proper reporting procedures are vital. (IX) Collaboration between dairy producers, veterinarians, and regulatory agencies should be fostered to share information and best practices. (X) Lastly, developing and regularly updating farm-specific contingency plans for managing potential HPAI outbreaks is essential to ensure preparedness and effective response.

By implementing these enhanced surveillance and biosecurity measures, the dairy industry can significantly reduce the risk of HPAI H5N1 introduction and spread, protecting both animal and human health. Ongoing research, vigilance, and strategy adaptation will be crucial as the situation continues to evolve.

### Future research priorities and ongoing surveillance needs

The emergence of HPAI H5N1 in dairy cattle presents a complex challenge that requires balancing trade interests, animal welfare, and public health priorities. The dairy industry’s economic significance necessitates careful consideration of control measures, as culling infected herds (which are economically valuable and more difficult to cull than poultry flocks) or implementing movement restrictions can lead to substantial financial losses and supply chain disruptions [114]. The current trade landscape is fraught with challenges, with at least 22 US states imposing new import requirements or restrictions on cattle. To maintain safe trade, a two-pronged approach is necessary: exporting countries must provide certification demonstrating compliance with science-based standards while importing countries should make risk-based decisions to allow for safe trade.

Animal welfare considerations are paramount in disease control efforts. Culling infected animals raises ethical concerns regarding livestock treatment and their high economic value, and public demand for humane animal treatment necessitates control measures aligned with these expectations. The psychological impact on farmers and workers involved in culling can lead to stress and emotional distress. Alternatives like vaccination, improved biosecurity measures, and enhanced surveillance should be prioritized to mitigate HPAI spread without resorting to drastic measures that compromise animal welfare and economic stability.

To address these multifaceted challenges, several policy recommendations are proposed. Enhanced biosecurity measures should be implemented across the dairy industry, particularly in high-risk areas. Exploring vaccination strategies for dairy cattle, engaging in discussions to harmonize trade protocols, and strengthening public health infrastructure are crucial steps. Additionally, investment in research to explore genetic resistance to avian influenza in cows and develop bovine-specific vaccines is essential. Clear, science-based communication strategies will help inform the public about the safety of dairy products, while targeted financial support can assist affected producers in managing economic losses.

## Lessons for pandemic preparedness and response: insights from the HPAI H5N1 outbreak in dairy cattle

### The importance of early detection and rapid containment of emerging zoonoses

The recent outbreak of HPAI H5N1 in dairy cattle underscores the critical need for early detection and rapid containment of emerging zoonoses [115, 116]. Historically, zoonotic diseases have posed significant threats to public health, agriculture, and economies worldwide. The swift identification of H5N1 cases in cattle and subsequent human infections highlights the importance of robust surveillance systems that can detect pathogens before they establish themselves in new hosts.

Early detection strategies are essential. (I) Comprehensive monitoring involves implementing routine testing of livestock and environmental samples. Utilizing genomic sequencing helps identify novel pathogens and track their evolution. Integrating veterinary health data with public health surveillance provides a holistic view of disease dynamics. (II) Surveillance systems require the establishment of robust mechanisms for routine livestock monitoring, conducting active surveillance through regular testing, and promptly investigating unusual disease syndromes or unexpected animal deaths. (III) Diagnostic infrastructure investment is crucial to handle large-scale testing during outbreaks and expanding diagnostic laboratory networks improves the timeliness of outbreak identification. Rapid, field-deployable assays can facilitate early detection at the point of need, improving outbreak response and control [11, 117].

Rapid containment measures are equally important. (I) Immediate biosecurity protocols should restrict the movement of infected animals, implement quarantine measures, and enhance PPE usage among farm workers. (II) A coordinated response involves developing joint contingency plans across public health, veterinary, and environmental sectors, conducting multi-sectoral simulation exercises, and designating liaison officers to facilitate communication between agencies. (III) Public awareness and education efforts engage agricultural workers and the general public in understanding zoonotic disease risks, providing training programs on hygiene, biosecurity practices, and PPE use.

Challenges and gaps in preparedness need addressing. (I) Insufficient diagnostic infrastructure for large-scale testing during outbreaks is a significant issue. (II) Inconsistent biosecurity practices across farms compromise containment efforts. (III) There is often a lack of coordination between animal and human health agencies. (IV) Inadequate surveillance of wildlife reservoirs remains a critical gap in comprehensive disease management.

Addressing these gaps requires increased funding, standardization of best practices, improved stakeholder coordination, and investment in innovative surveillance technologies.

### Strengthening collaboration between public health, veterinary, and agricultural sectors

**Fig. 4:**
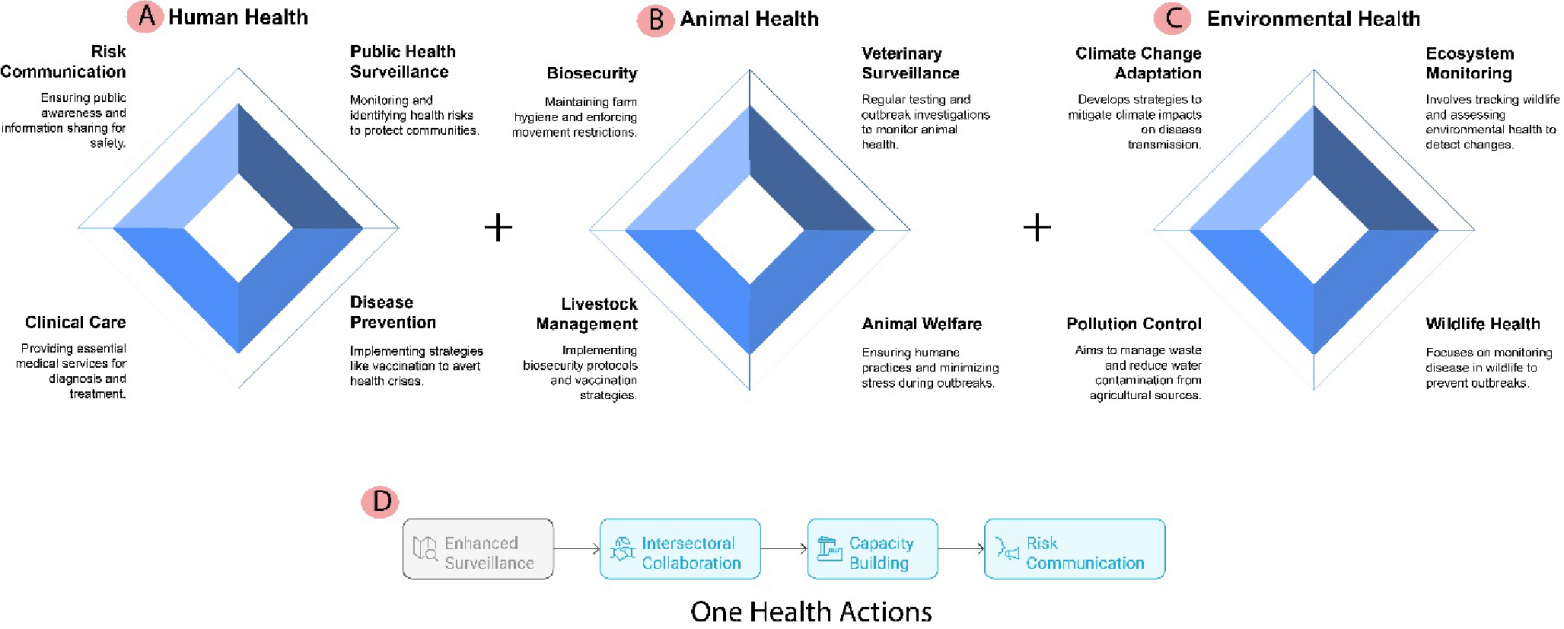
One Health Actions: Integrating Human, Animal, and Environmental Health

The emergence of HPAI H5N1 in dairy cattle highlights the critical need for enhanced collaboration between public health, veterinary, and agricultural sectors [54]. A One Health framework, integrating human, animal, and environmental health, is essential for fostering communication and cooperation among these sectors (Fig. 4) [54].

Collaborative approaches include several key strategies. (I) Establishing joint task forces and intersectoral committees is crucial for sharing vital information, resources, and expertise, thereby improving surveillance and response capabilities to emerging infectious diseases. (II) Developing integrated surveillance systems allows for monitoring zoonotic pathogens across various species and environments, providing a more holistic view of disease dynamics.

Educational initiatives play a significant role as well. III) Raising awareness about zoonotic diseases among farmers, veterinarians, and public health professionals is vital. Implementing training programs on biosecurity measures, disease recognition, and reporting protocols enhances preparedness.

Community partnerships are also important. IV) Building partnerships with local communities fosters grassroots engagement in surveillance and response efforts. Ensuring interventions are culturally appropriate and effectively implemented boosts their success.

This figure presents a framework for One Health actions, highlighting the interconnected roles of human, animal, and environmental health and the essential strategies for improving overall health outcomes.

### Key areas for strengthening multi-sectoral cooperation

Key areas for strengthening multi-sectoral cooperation are crucial for addressing challenges like HPAI H5N1 in dairy cattle. Integrated surveillance and diagnostic capacity are essential components [54]. Investing in integrated human-animal disease surveillance systems will enhance early detection and response capabilities. Additionally, providing joint training for public health and veterinary professionals on surveillance and diagnostic methods ensures cohesive operations and improves overall effectiveness.

Coordinated outbreak response is another critical area that requires attention [54]. Developing clear coordination mechanisms between public health, veterinary, and agricultural agencies can streamline outbreak management. Conducting multi-sectoral simulation exercises further enhances readiness and fosters collaboration during actual events.

Standardized biosecurity measures are also vital [116]. Collaborating with industry to develop practical biosecurity guidelines tailored to specific needs will strengthen preventive efforts. Offering training and resources supports effective implementation at the farm level. Furthermore, utilizing technology, such as IoT sensors for real-time monitoring of livestock health, can significantly enhance preventive measures [118].

Targeted research and development should be prioritized to address specific challenges. Allocating funding towards research on the genetic resistance of cattle to avian influenza can inform breeding programs. Focusing on developing cattle-specific influenza vaccines will help mitigate disease impact. Establishing public-private partnerships encourages innovation in vaccine development and biosecurity technologies.

Finally, risk communication and community engagement play a crucial role. Formulating joint risk communication strategies with consistent messaging across sectors ensures clear public understanding. Engaging local communities, particularly farm workers, through tailored education programs emphasizes biosecurity and disease prevention practices, ultimately contributing to more effective risk management.

### Challenges and recommendations

Challenges and recommendations in addressing the emergence of HPAI H5N1 in dairy cattle are critical for improving multi-sectoral cooperation. One of the primary challenges is ensuring political will and sustained investment. It is essential to prioritize funding for integrated surveillance systems, joint capacity building, and targeted research efforts. Additionally, forging strategic partnerships with industry and international organizations can help mobilize the necessary resources and expertise to address these challenges effectively.

Another significant challenge is breaking down silos between sectors. Encouraging cross-sectoral collaboration and information sharing is vital for a more unified approach to disease management. Developing mechanisms for regular communication and joint decision-making among different sectors will enhance collaboration and ensure that all parties are aligned in their efforts.

Capacity building is also a crucial aspect of overcoming these challenges. Investing in training programs that foster a One Health approach among professionals in all sectors will help create a more integrated workforce. Furthermore, developing curricula that integrate public health, veterinary science, and agricultural studies will prepare future professionals to work collaboratively in addressing zoonotic diseases.

By addressing these challenges and implementing the recommended strategies, we can strengthen the response to HPAI H5N1 and enhance overall public health security.

## Conclusion

### Recapitulation of key points

The emergence of HPAI H5N1 in dairy cattle marks a significant shift in the epidemiology of zoonotic influenza viruses, highlighting the interconnectedness of animal, human, and environmental health. This outbreak has revealed critical vulnerabilities in our current surveillance and response systems, particularly in the context of livestock management and public health. The rapid spread of H5N1 across multiple states in the US, coupled with confirmed human cases linked to occupational exposure, underscores the urgent need for enhanced monitoring and biosecurity measures within the agricultural sector. Furthermore, the unique pathogenesis of H5N1 in dairy cattle, characterized by high viral loads in milk and the potential for zoonotic transmission, necessitates a reevaluation of existing public health strategies to mitigate risks associated with this emerging threat.

### The urgent need for a proactive and integrated one health approach to zoonotic influenza

The complexities of zoonotic disease transmission, particularly in the context of HPAI H5N1, necessitate a proactive and integrated One Health approach that encompasses human, animal, and environmental health sectors. This framework is essential for understanding the dynamics of disease emergence and for implementing effective prevention and control strategies. The One Health model promotes collaboration among veterinarians, public health officials, and environmental scientists, facilitating the sharing of data and resources to enhance surveillance and response capabilities. By prioritizing a One Health approach, stakeholders can better address the multifaceted challenges posed by zoonotic diseases, ensuring that interventions are comprehensive and context-specific. This integrated strategy is particularly crucial in regions with high livestock density and close human-animal interactions, where the risk of spillover events is heightened.

### Call to action for policymakers, researchers, and health professionals to prioritize this emerging threat

In light of the ongoing HPAI H5N1 outbreak and its implications for public health, it is imperative that policymakers, researchers, and health professionals take immediate action to prioritize this emerging threat. Policymakers must allocate resources for enhanced surveillance systems that monitor both avian and mammalian populations, ensuring that potential outbreaks are detected early. Additionally, investment in research to understand the epidemiology and pathogenesis of H5N1 in cattle and other mammals is essential for developing targeted vaccines and therapeutics. To prepare for potential pandemics from avian flu viruses, countries have stockpiled vaccines. It is crucial to test these vaccines against emerging strains like H5N1 in livestock. The World Health Organization (WHO) has selected two candidate vaccine viruses (CVVs) for the H5N1 clade 2.3.4.4b: IDCDC-RG78A and NIID-002 [119, 120]. Urgent evaluation of their efficacy against viral strains in animals is needed. Health professionals should advocate for the implementation of robust biosecurity measures on farms and promote education and training for agricultural workers to reduce occupational exposure risks. By fostering a collaborative environment that emphasizes the importance of One Health, stakeholders can effectively mitigate the risks associated with HPAI H5N1 and other zoonotic diseases, ultimately safeguarding public health and ensuring the sustainability of agricultural practices.

Finally, the emergence of HPAI H5N1 in dairy cattle serves as a critical reminder of the need for vigilance and preparedness in the face of evolving zoonotic threats. The integration of efforts across sectors, coupled with a commitment to proactive disease management, will be essential in addressing the challenges posed by this and future outbreaks.

## Data Availability

No datasets were generated or analysed during the current study.
